# Fluidodinâmica Computacional na Avaliação do Risco Futuro de Aneurismas de Aorta Ascendente

**DOI:** 10.36660/abc.20200926

**Published:** 2022-02-14

**Authors:** Gabriela de C. Almeida, Bruno Alvares de Azevedo Gomes, Fabiula Schwartz de Azevedo, Karim Kalaun, Ivan Ibanez, Pedro S. Teixeira, Ilan Gottlieb, Marcelo M. Melo, Glaucia Maria Moraes de Oliveira, Angela O. Nieckele

**Affiliations:** 1 Departamento de Engenharia Mecânica Pontifícia Universidade Católica do Rio de Janeiro Rio de Janeiro RJ Brazil Departamento de Engenharia Mecânica, Pontifícia Universidade Católica do Rio de Janeiro, Rio de Janeiro, RJ – Brazil; 2 Departamento de Cardiologia Universidade Federal do Rio de Janeiro Rio de Janeiro RJ Brazil Departamento de Cardiologia, Universidade Federal do Rio de Janeiro, Rio de Janeiro, RJ – Brazil; 3 FIT Center Niterói RJ Brazil FIT Center – Clínica de Performance Humana, Niterói, RJ – Brazil; 4 Casa de Saúde São José Rio de Janeiro RJ Brazil Casa de Saúde São José, Rio de Janeiro, RJ – Brazil; 5 Instituto Nacional de Cardiologia Rio de Janeiro RJ Brazil Instituto Nacional de Cardiologia, Rio de Janeiro, RJ – Brazil

**Keywords:** Hemodinâmica, Aneurisma Aórtico, Aneurisma da Aorta Torácica, Modelagem Computacional Específica para o Paciente

## Abstract

**Fundamentos:**

Uma metodologia para identificação de pacientes portadores de aneurisma de aorta ascendente (AAAs) sob alto risco de remodelamento aórtico não está completamente definida.

**Objetivo:**

Esta pesquisa objetiva caracterizar numericamente o fluxo sanguíneo aórtico, relacionando a distribuição do estresse mecânico resultante com o crescimento de AAAs.

**Métodos:**

Estudo analítico, observacional, unicêntrico, em que um protocolo de fluidodinâmica computacional (CFD - Computacional Fluid Dynamics) foi aplicado a imagens de angiotomografia computadorizada (ATC) de aorta de pacientes portadores de AAAs. Duas ATC de aorta com pelo menos um ano de intervalo foram obtidas. Dados clínicos dos pacientes foram registrados e, a partir das imagens de ATC, foram gerados modelos tridimensionais. Foram realizados estudos do campo de velocidade e estruturas coerentes (vórtices) com o objetivo de relacioná-los ao crescimento ou não do aneurisma e, posteriormente, compará-los com os dados clínicos dos pacientes. O teste de Kolmogorov-Smirnov foi utilizado para avaliar a normalidade da amostra e o teste não-paramétrico Wilcoxon signed-rank foi aplicado para comparações de dados pareados entre os ângulos aórticos. A significância estatística foi fixada em 5%.

**Resultados:**

Para o grupo que apresentou crescimento do aneurisma, a incidência do jato na parede aórtica gerou áreas de recirculação posterior ao jato, induzindo à formação de vórtices complexos, ocasionando um incremento na pressão média no endotélio aórtico. O grupo sem crescimento do aneurisma apresentou diminuição na pressão média.

**Conclusão:**

Este estudo piloto mostrou que a CFD baseada em ATC pode, em um futuro próximo, ser uma ferramenta auxiliar na identificação dos padrões de fluxo associados ao processo de remodelamento de AAAs.

## Introdução

Aneurisma de aorta ascendente (AAAs) é geralmente assintomático e sua evolução é imperceptível.^1 , 2^ Entretanto, suas complicações, como ruptura e dissecção, frequentemente são eventos catastróficos.^3^ Alguns aneurismas não crescem, enquanto outros têm um aumento significativo de suas dimensões em um curto período de tempo. As variáveis relacionadas ao crescimento do AAAs ainda não são perfeitamente compreendidas.^2 , 4^

A formação do AAAs é um processo degenerativo multifatorial, resultante de fatores hemodinâmicos e processos biológicos.^5^ Segundo Hope et al.,^4^ mudanças no comportamento do fluxo ao longo da aorta ascendente são relacionadas ao processo de remodelamento e, portanto, podem influenciar no crescimento aneurismático.

Cada vez mais, a fluidodinâmica computacional (CFD) tem se tornado uma ferramenta complementar para promover o melhor entendimento da patogênese e progressão de doenças cardiovasculares, além de se mostrar adequada para avaliações minimamente invasivas.^6 , 7^ Como destacado por Morris et al.,^7^ modelos de CFD aplicados em escala populacional têm o potencial de reduzir riscos, custos e tempo associados aos estudos clínicos.

Através de estudos de CFD do fluxo aórtico, é possível identificar regiões da parede aórtica com altos valores de tensão de cisalhamento e de pressão,^8^ sugerindo uma associação dessas grandezas com a dilatação aneurismática.^9^ Essas observações foram discutidas por Gülan et al.,^10^ que encontraram fluxos rotacionais formados na fase sistólica, mostrando similaridade qualitativa com a formação de anel de vórtice, isto é, um jato central cercado por dois grandes vórtices. Sob o ponto de vista da mecânica dos fluidos, o fluxo através de uma expansão abrupta, como ocorre entre o anel aórtico e a porção dilatada da aorta ascendente, leva à separação da camada limite e à formação de uma zona de separação. Portanto, é possível que haja uma correlação entre separação do fluxo, turbulência, variação de pressão e crescimento do aneurisma.^10^

**Figura 1:**
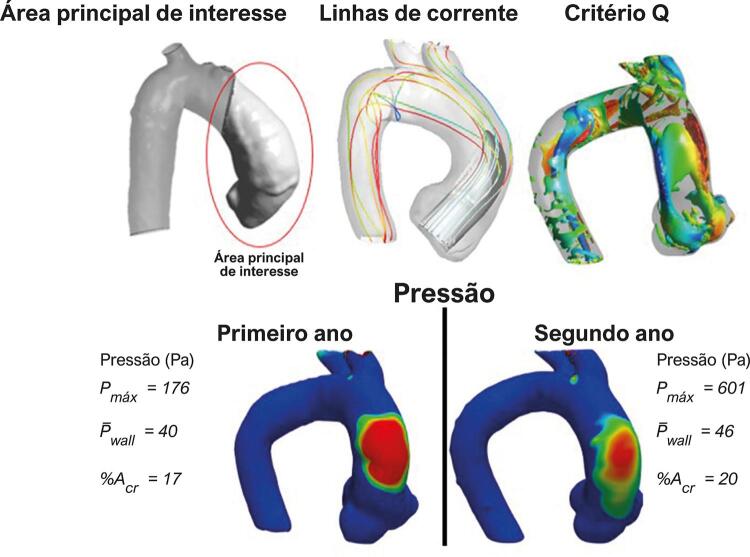
– Área principal de interesse da aorta ascendente, com principais variáveis de fluxo.

A presença de estruturas coerentes dentro da aorta também pode explicar a formação de regiões com altas tensões cisalhantes e altas pressões, o que pode contribuir com o processo de remodelamento aórtico.^11^ A Figura 1 mostra a região da aorta ascendente de interesse com as principais variáveis de fluxo, sendo a ilustração central do presente estudo. O objetivo deste estudo piloto é investigar o comportamento do escoamento através da aorta ascendente, visando identificar alguns padrões de fluxo que poderiam estar associados com o crescimento dos AAAs.

## Métodos

### Diretrizes do estudo

O presente estudo é analítico, observacional, unicêntrico, no qual um protocolo de CFD foi aplicado a imagens de angiotomografia computadorizada (ATC) de aorta. A pesquisa foi registrada na Comissão Nacional de Ética em Pesquisa do Ministério da Saúde e aprovada pelo comitê de ética em pesquisa local (CAAE 86716318.3.0000.5272, número: 2.750.919). Foi obtido consentimento informado de todos os participantes, de acordo com a resolução 466/2012 do Conselho Nacional de Saúde.

### Pacientes

Para realização do estudo, nove pacientes com AAAs foram investigados, tendo sido selecionados a partir de avaliações de 100 pacientes consecutivos do ambulatório de aortopatias de um centro terciário de saúde. Pacientes com diagnóstico de AAAs foram incluídos e aqueles com doenças do colágeno, cirurgia cardíaca prévia ou de aorta foram excluídos. Para todos os pacientes, duas imagens de ATC, com um intervalo mínimo de um ano entre os exames, encontravam-se disponíveis. As imagens foram obtidas por indicações clínicas de acompanhamento, não especificamente para o presente estudo. Os pacientes estavam sob tratamento clínico, de acordo com as diretrizes recentes sobre AAAs.^12^

### Geometria: segmentação e criação do modelo tridimensional

A geometria de cada aorta foi construída com base em uma varredura de ATC obtida por um scanner de 64 canais SOMATOM Sensation® 64 (Siemens®, Alemanha). Os cortes selecionados de ATC foram medidos desde o anel aórtico até a aorta descendente. Informações como tamanho de pixel e distância dos planos de aquisição das imagens foram adquiridos a fim de ajustar o modelo aórtico tridimensional (3D) ao seu tamanho real. A segmentação da imagem foi realizada através do programa FIJI de processamento de imagem de código aberto baseado em ImageJ, focado em análise de imagens biológicas.^13^

**Figura 2:**
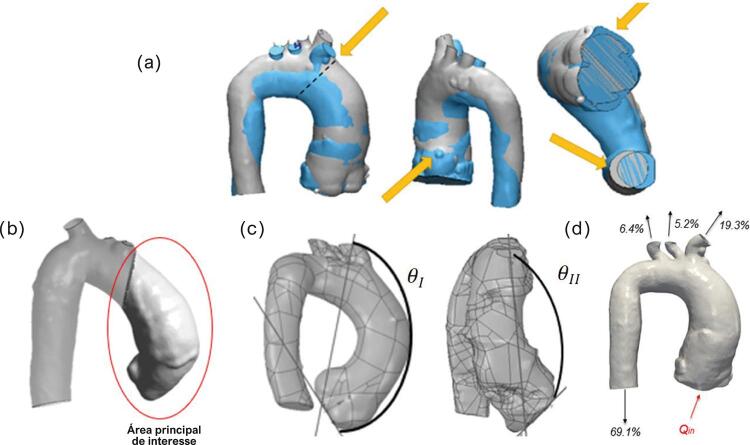
– (a) Critérios de comparação entre aortas do mesmo paciente em diferentes anos. A seta amarela indica superposição dos troncos braquiocefálicos, com a linha pontilhada indicando seu início, e sobreposição das artérias coronárias direitas das aortas, sendo cortadas as valvas aórticas e as porções descendentes das aortas; (b) Área principal de interesse das aortas; (c)θ_I_ e θ_II_; (d) Condições de entrada e saída.

Dois modelos aórticos 3D de cada paciente foram sobrepostos visando obter referência espacial e possibilitar a comparação entre os exames, através da superposição do início do tronco braquiocefálico e das artérias coronárias direitas ( Figura 2a ). A aorta correspondente ao primeiro ano de exame está colorida de cinza e a do ano posterior, de azul. Uma vez que as estruturas se encontravam sobrepostas, a valva aórtica e a parte descendente da aorta foram cortadas ( Figura 2a ), garantindo a mesma referência espacial para as seções de entrada e saída do fluxo sanguíneo. A Figura 2b mostra a principal área de interesse do estudo, a aorta ascendente. O plano de entrada do fluxo na aorta foi posicionado no centroide do anel aórtico, de modo que o eixo x cruzasse o centroide do tronco coronariano esquerdo, apontando para a parede anterior da aorta, e o eixo y apontasse para a artéria coronária direita.

Uma vez que o campo de escoamento depende diretamente da anatomia aórtica, dois ângulos ( *θ*_*I*_ e *θ*_*II*_ ) foram definidos entre o plano de entrada e a saída do tronco braquiocefálico, com o objetivo de relacioná-los com o padrão de fluxo que pode induzir o crescimento de um aneurisma ( Figura 2c ). *θ*_*I*_ corresponde ao ângulo formado entre a linha que conecta o centroide do tronco braquiocefálico com o centroide do tronco coronariano esquerdo e o eixo x centralizado no plano de entrada. *θ*_*II*_ corresponde ao ângulo entre a linha que conecta o centroide do tronco braquiocefálico com um ponto localizado na posição extrema do eixo x do plano de entrada e a linha que conecta esse ponto com um ponto na extremidade do eixo y do plano de entrada. A Figura 2d ilustra a entrada da valva aórtica e as quatro seções de saída.

### Modelagem do escoamento

A análise da correspondência do campo de escoamento com a patologia do paciente foi realizada focando na porção ascendente da aorta, durante a sístole ventricular, quando as paredes da aorta se encontram distendidas. Durante o pico sistólico, ocorre o máximo diâmetro da aorta, que ao apresentar baixa complacência, permite considerar a parede aórtica como rígida.^14^ Adicionalmente, a presente análise foi realizada para a situação mais crítica durante a sístole, correspondendo à máxima vazão fisiológica, *Q*_*in*_ = 25 l/min,^15^ considerando regime turbulento estatisticamente permanente.^16^ Para modelar a turbulência, o modelo de média de Reynolds de duas equações κ−ω SST^17^ foi selecionado, baseado na recomendação de Celis et al.,^18^ que realizaram uma comparação numérica com os dados experimentais de Gomes et al.^19^ em uma anatomia aórtica similar. Os efeitos da gravidade também foram negligenciados, visto que as variações de pressão no interior da aorta são dominantes sobre efeitos gravitacionais.

Uma vez que são encontradas altas taxas de cisalhamento^20^ em pacientes com aneurisma de aorta ascendente, o sangue foi modelado como um fluido Newtoniano (aproximação válida para taxas de deformação acima de 50 s^-1^).^21^ Em condições normais, o sangue pode ser considerado um fluido incompressível,^22^ tendo a densidade sido fixada em *ρ* = 1054 kg/m^3^ A viscosidade dinâmica foi definida como *μ* = 7,2 cP para permitir uma comparação direta com os dados experimentais *in vitro* .^18 , 19 , 23^ Adicionalmente, foram realizados alguns testes e os resultados obtidos com viscosidades iguais a 3,5 cP e 7,2 cP forneceram resultados equivalentes, com diferenças de pressão inferiores a 0,01 mmHg na região de interesse da aorta ascendente. Esses testes corroboram os achados de Becsek et al.,^24^ que avaliaram viscosidades iguais a 4 cP, 6 cP e 8 cP e mostraram efeito desprezível no campo de escoamento médio e efeito muito pequeno com relação a transição turbulenta.

Para todos os casos, as mesmas distribuições de vazões nas saídas (porcentagens baseadas no fluxo de entrada) foram impostas com base em valores médios do corpo humano, em acordo com Alastruey et al.,^15^ conforme mostrado na Figura 2d : aorta descendente, 69,1%; tronco braquiocefálico, 19,3%; artéria carótida esquerda, 5,2%; e artéria subclávia esquerda, 6,4%.

### Simulação numérica

A presente análise foi realizada utilizando-se o programa ANSYS *Fluent* v18.1, que resolve as equações de conservação discretizadas com base no método de volumes finitos.^25^ As simulações foram pós-processadas com a ferramenta ANSYS CFD- *Post tool* ^26^ e com o programa de código aberto Paraview.^27^

As equações discretizadas foram obtidas com o esquema “ *power-law* ”,^25^ o acoplamento pressão-velocidade foi tratado pelo algoritmo SIMPLE^25^ e o erro residual máximo de convergência foi definido em 10^-6^ para todas as equações.

Para todos os casos estudados, empregaram-se 400.000 nós, os quais foram definidos após a realização de um teste de independência da solução na malha. Garantiram-se variações inferiores a 0,3% na queda de pressão na área principal de interesse (aorta ascendente), ao dobrar a malha.

### Análise estatística

A forma anatômica da aorta de cada paciente pode ser inferida pelos ângulos de referência *θ*_*I*_ e *θ*_*II*_ ( Figura 2c ), que foram medidos de forma independente por dois observadores. Variáveis contínuas com distribuição normal foram apresentadas como média e desvio padrão. Variáveis contínuas sem distribuição normal foram apresentadas como mediana e intervalo interquartil. A reprodutibilidade inter-observador foi calculada pelo coeficiente de correlação intraclasse (CCI) para as medidas de *θ*_*I*_ e *θ*_*II*_ . O seguinte critério de confiabilidade^28^ foi aplicado: pobre (CCI ≤ 0,50); moderada (0,50 < CCI ≤ 0,75); bom (0,75 < CCI ≤ 0,90); excelente (CCI > 0,90).

O teste de normalidade Kolmogorov-Smirnov foi usado para examinar se as variáveis apresentavam distribuição normal. Devido à distribuição não normal, o teste não paramétrico Wilcoxon *signed-rank* , foi aplicado para comparações de dados pareados (amostras dependentes) entre as medições de *θ*_*I*_ e *θ*_*II*_ . A significância estatística foi estabelecida em 5%. Todas as análises estatísticas foram conduzidas utilizando-se o software da IBM® SPSS Statistics® versão 26.^29^

## Resultados

### Formato aórtico e ângulos

Os pacientes foram divididos em dois grupos de acordo com o volume da região de interesse.^30^ Considerou-se que o aneurisma cresceu, quando se observou um aumento de 10% ou mais no volume da região de interesse, ao comparar as duas ATC de cada paciente. A Tabela 1 apresenta o percentual da diferença de volume e o intervalo de tempo entre as tomografias de cada paciente. Não houve diferenças estatísticas entre as medidas dos dois observadores independentes. O CCI resultante foi de 0,99, considerado excelente.

**Tabela 1:** – Classificação do paciente de acordo com o crescimento do aneurisma

	Paciente	Variação do volume (%)	Intervalo entre exames (anos)	Sexo	Idade (anos)	Ano	*θ*_***I***_ (º)	*θ*_***II***_ (º)
**Aneurisma com crescimento**	1	18,0	2	M	77	1	138,80	63,56
						2	144,85	56,61
	2	10,0	1	F	60	1	116,64	51,34
						2	94,74	42,69
	3	15,6	2	M	70	1	116,51	42,61
						2	126,91	46,07
	4	10,5	3	M	63	1	124,14	51,71
						2	128,03	51,59
	5	12,5	2	M	58	1	135,79	51,30
						2	134,74	67,86
**Aneurisma sem crescimento**	6	0,50	1	F	63	1	94,90	78,84
						2	88,33	91,39
	7	-4,50	2	F	52	1	77,19	70,86
						2	94,20	71,99
	8	-0,13	2	M	74	1	119,22	64,07
						2	121,53	56,89
	9	-0,20	3	M	69	1	136,41	66,14
						2	137,09	58,99

*M: masculino; F: feminino.*

Conforme mostrado na Tabela 1 , a variação de volume dos pacientes 6, 8 e 9 é desprezível. Baseado na diferença entre a medida do volume aórtico, pacientes com variação de volume igual ou superior a 10% (pacientes 1 a 5) foram considerados como tendo aumento do aneurisma e, abaixo desse valor limiar (pacientes 6 a 9), o aneurisma foi considerado como não apresentando crescimento. A mediana da variação de volume e o intervalo interquartil em pacientes com crescimento do aneurisma foi de 12,5 [10,25 – 16,8]%, naqueles sem crescimento do aneurisma foi de (-0,16) [(-2,35) – (0,18)]% e na população total foi de 10 [(-0,16) – (14,05)]%.

As medidas correspondentes ao primeiro e ao segundo ano estão listadas na Tabela 1 , para todos os pacientes, onde se pode observar que aqueles identificados com crescimento de aneurisma apresentaram grande *θ*_*I*_ ( *θ*_*I*_ *≥* 94^o^) e pequeno *θ*_*II*_ (51^o^ *≤ θ*_*II*_ *≤* 68^o^). Entretanto, ao examinar os ângulos correspondentes aos pacientes sem crescimento do aneurisma, não se observou uma tendência nítida. Existem pacientes com grandes e pequenos *θ*_*I*_ e *θ*_*II*_ . Os pacientes 6 e 7 apresentaram claramente uma faixa diferente de ângulos, contudo, o mesmo não é verdade para os pacientes 8 e 9. A medição de *θ*_*I*_ em pacientes com crescimento de aneurisma foi de 127,47 [116,64 – 135,79]°, naqueles sem crescimento do aneurisma foi de 119,22 [91,26 – 128,97]° e na população total foi de 121,53 [94,9 – 135,79]°. As medições de *θ*_*II*_ em pacientes com crescimento de aneurisma foi de 51,46 [46,07 – 56,61]°, naqueles sem crescimento de aneurisma foi de 68,5 [61,53 – 75,41]° e da população total foi 57,94 [51,34 – 67,86]°.

### Padrões do fluxo sanguíneo

**Figura 3:**
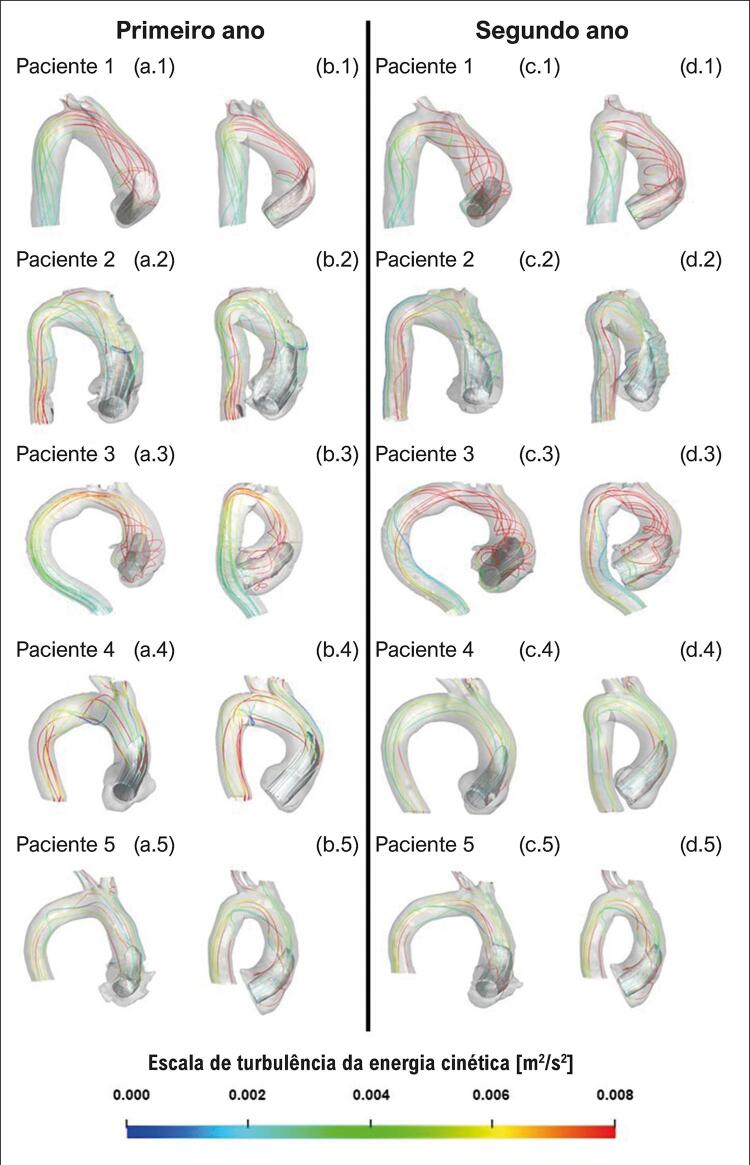
– Iso-superfície de velocidade axial (w/w_in_ = 0,5) e linhas de corrente coloridas de acordo com a escala da energia cinética turbulenta. Pacientes com crescimento do aneurisma: vista frontal (a) e vista lateral (b) do primeiro ano; vista frontal (c) e vista lateral (d) do segundo ano.

**Figura 4:**
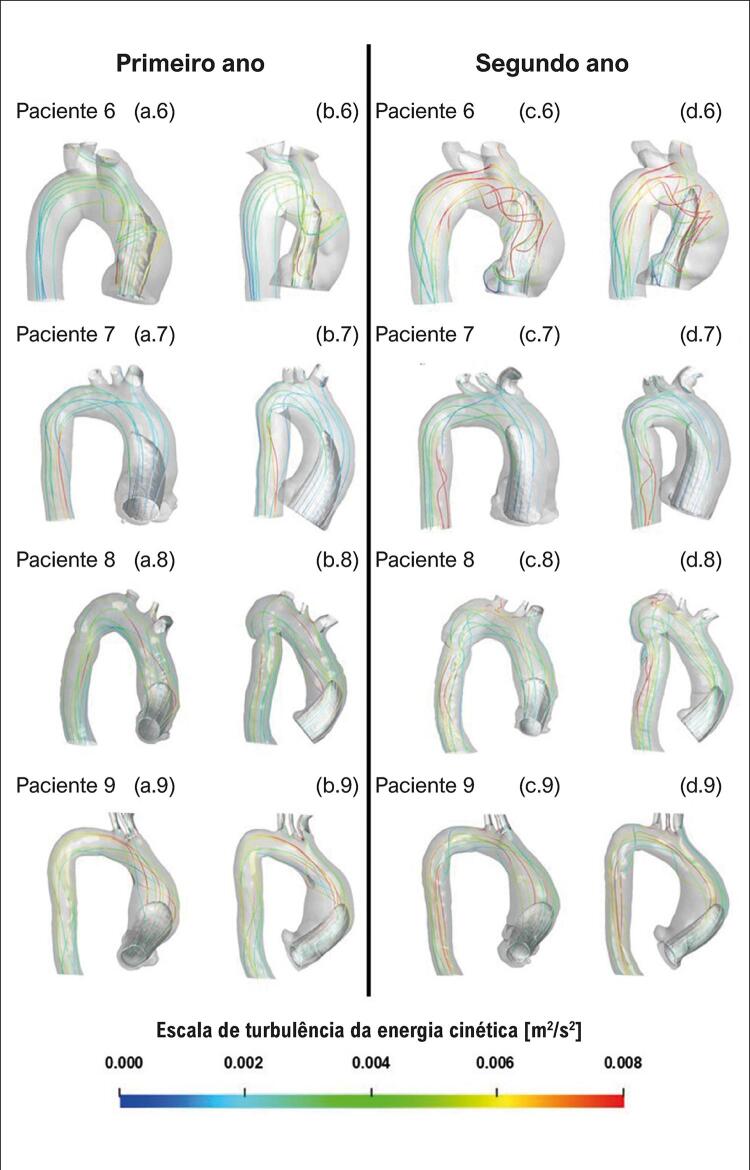
– Iso-superfície de velocidade axial (w/w_in_ = 0,5) e linhas de corrente coloridas de acordo com a escala da energia cinética turbulenta. Pacientes sem crescimento do aneurisma: vista frontal (a) e vista lateral (b) do primeiro ano; vista frontal (c) e vista lateral (d) do segundo ano.

Para visualizar o campo de escoamento no interior da aorta, empregando-se vistas frontal e lateral da aorta, traçou-se uma iso-superfície cinza correspondente a velocidade axial ( *w* ) igual a 50% da velocidade axial de entrada ( *w*_*in*_ ) ( Figuras 3 e 4 correspondem a pacientes com e sem crescimento de aneurisma, respectivamente). Essa variável permite a identificação do formato do jato de entrada. Linhas de corrente também estão incluídas nas figuras para auxiliar na identificação de recirculações. Para avaliar o nível de turbulência, essas linhas foram coloridas de acordo com a energia cinética turbulenta, κ. Analisando a Figura 3 , para todos os cinco pacientes com crescimento de aneurisma, o jato de entrada é direcionado para a parede aórtica anterior. Além disso, podem ser observadas grandes regiões rotacionais, exceto para o paciente 4, que, como será mostrado mais adiante, apresentou aumento substancial do nível de pressão no endotélio vascular. Esses resultados corroboram as observações feitas por Gülan et al.^10^ que sugeriram que a presença de regiões rotacionais possa estar associada ao crescimento do aneurisma. Nos pacientes 6 e 7, cujo aneurisma não cresceu ( Figura 4 ), as linhas de corrente resultantes se estendem pela aorta, visto que o jato não incide diretamente na parede, pois *θ*_*I*_ ( Tabela 1 ) é menor. Embora jatos de entrada mais longos também possam ser observados para os pacientes 8 e 9 quando comparados com os pacientes 1 a 5, o jato de entrada também colide sobre a parede anterior, devido ao seu grande *θ*_*I*_ , mas nenhuma região de recirculação é observada, ou seja, as linhas de corrente seguem um caminho suave ao longo da anatomia aórtica. Vale salientar que o campo de escoamento do paciente 6, cujo aneurisma não cresceu, mostra regiões de recirculação, mas essas são localizadas numa região acima da aorta ascendente. Em relação ao nível de turbulência, não foram observadas diferenças significativas para ambos os grupos. Para todos os casos, foi imposto 5% de intensidade turbulenta na entrada da valva aórtica e, para ambos os grupos, obteve-se uma intensidade média de turbulência entre 1% e 2%.

Segundo Weigang et al.,^31^ alguns casos com crescimento aneurismático apresentam a formação de grandes regiões rotacionais, com um anel de vórtice circundando um jato central com dois grandes vórtices. Assim, para identificar estruturas coerentes no escoamento, foi empregado o critério *Q* ,^32^ definido como


$$Q=\left(\Omega_{ij}\Omega_{ij}-S_{ij}S_{ij};S_{ij}=\frac{1}{2}\left(\frac{\partial u_{i}}{\partial x_{j}}+\frac{\partial u_{j}}{\partial x_{i}}\right);\Omega_{ij}=\frac{1}{2}\left(\frac{\partial u_{i}}{\partial x_{j}}-\frac{\partial u_{j}}{\partial x_{i}}\right)\right.$$ (1)

onde um *Q* positivo significa que a magnitude do tensor vorticidade *Ω* é dominante em relação a taxa de deformação *S* . Além disso, *Q* é relacionado a regiões de baixa pressão, que também podem estar associadas à presença de estruturas coerentes. Adicionalmente, foi calculada a helicidade normalizada^33^


$$H=\xi_{i}u_{i}/\left[\sqrt{\xi_{k}\xi_{k}}\sqrt{u_{\ell }u_{\ell }}\right];\xi_{i}=\epsilon_{ijk}\partial u_{j}/\partial u_{i}$$ (2)

onde *ξ*_*i*_ é a vorticidade e *ϵ*_*i* *jk*_ , o operador Levi-Civita. A helicidade avalia a tendência do escoamento em formar vórtices coerentes, representando a quantidade de ligação das linhas de vórtices do fluxo.^34^ De acordo com Garcia et al.,^35^ o fluxo helicoidal pode promover dilatação aórtica, portanto, a helicidade normalizada pode auxiliar na caracterização de doenças valvares e cardiopatias. Ademais, quando as direções da vorticidade e do vetor velocidade são coincidentes, a helicidade normalizada é *H = 1* , quando são opostas, *H = –1* e para vetores ortogonais *H = 0* . Assim, a helicidade é uma indicação quantitativa do fluxo helicoidal em torno da direção do fluxo principal na região aórtica.^35^ Os valores de helicidade de 1 ou −1 representam o núcleo do vórtice, que é caracterizado por forte enrolamento do vórtice. Gallo et al.^36^ indicam que um valor de helicidade normalizado absoluto superior a 0,6 representa a possibilidade de um remodelamento vascular, mas Garcia et al.^35^ indicam um limiar ligeiramente maior, 0,8.

**Figura 5:**
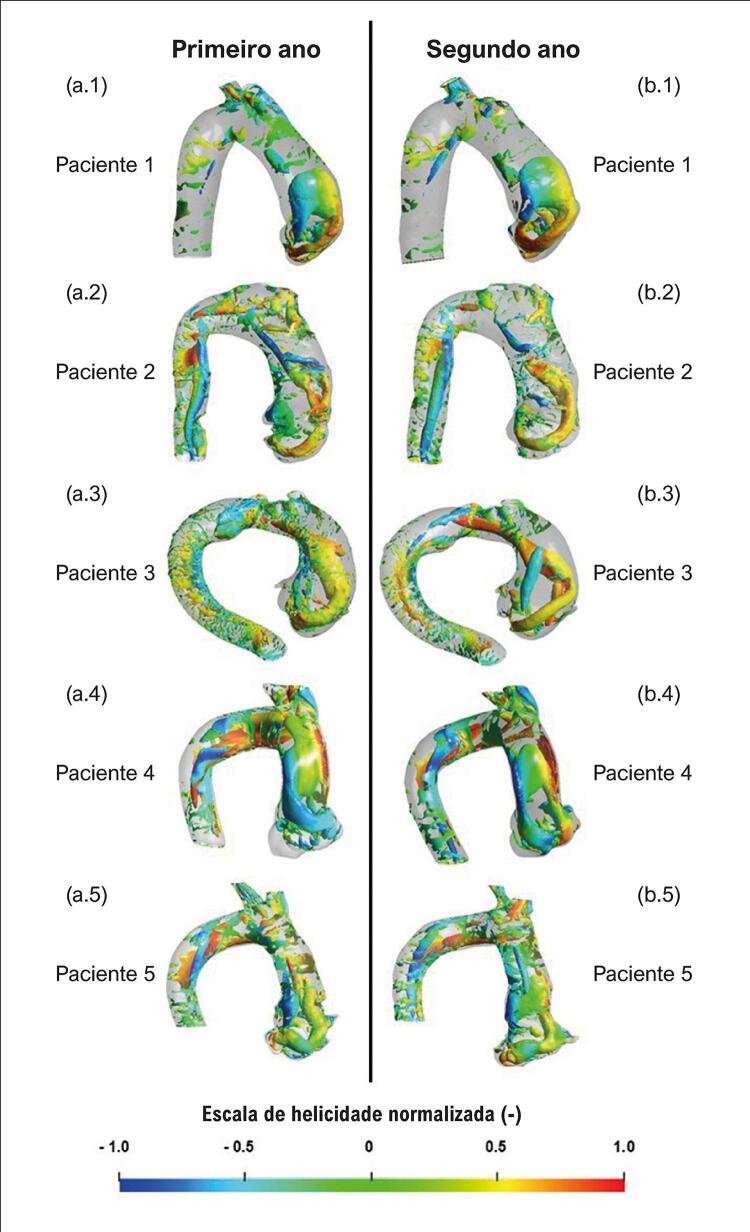
– Iso-superfície Q, colorida de acordo com a escala de helicidade normalizada. Pacientes com crescimento de aneurisma: (a) primeiro ano; (b) segundo ano.

Uma iso-superfície *Q* para cada paciente, normalizada pela velocidade de entrada e diâmetro efetivo, é traçada nas Figuras 5 e 6 , correspondendo aos dois grupos, respectivamente. Cada iso-superfície *Q* foi colorida com a helicidade normalizada *H* , com a escala apresentada na parte inferior das figuras.

**Figura 6:**
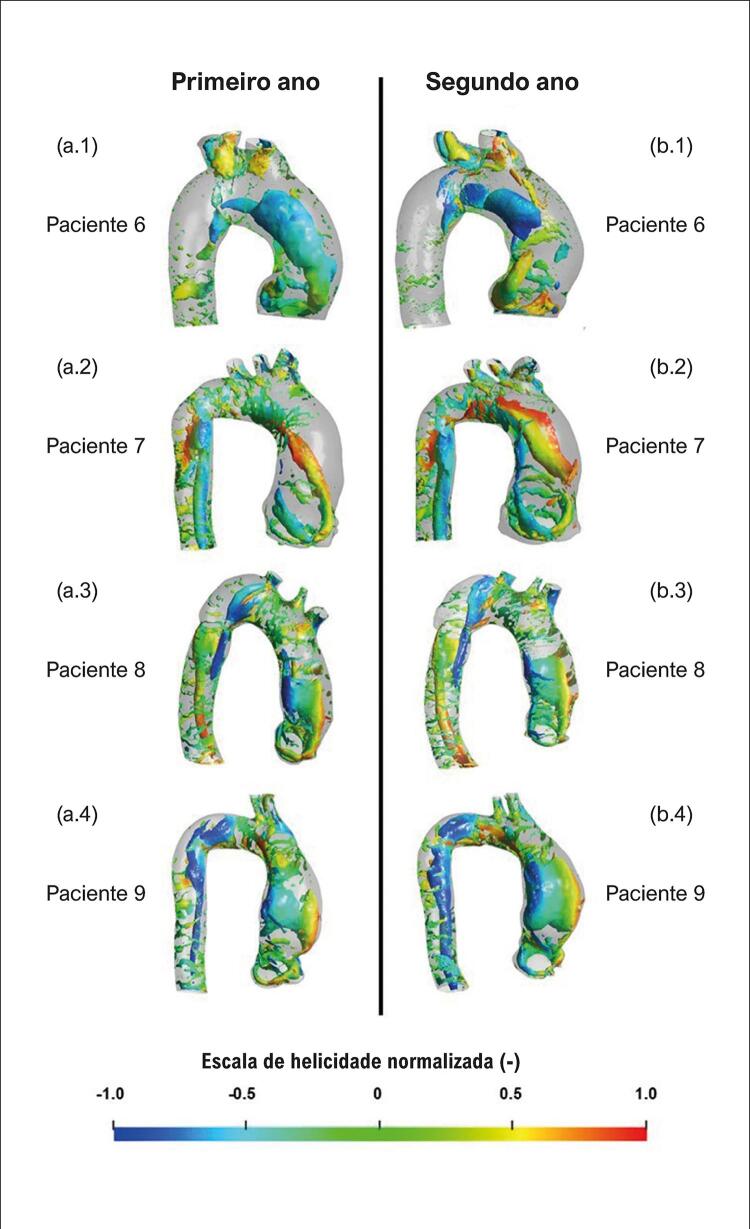
– Iso-superfície Q, colorida de acordo com a escala de helicidade normalizada. Pacientes sem crescimento de aneurisma: (a) primeiro ano; (b) segundo ano.

Analisando a Figura 5 para o paciente 1, que apresenta a maior dilatação aórtica, pode-se observar uma grande estrutura de vórtice com formato semelhante a um vórtice *hairpin* (i.e., em forma de grampo de cabelo) atingindo a parede distal, em ambos os anos. A curvatura angular do paciente 1 pode ter causado a formação do vórtice descolado. O paciente 2 (menor dilatação do grupo) apresentou um vórtice toroidal próximo à raiz da aorta, com um vórtice longitudinal ao longo da parede distal. O mesmo fenômeno foi observado para os pacientes 3 e 5. Examinando a iso-superfície *Q* para o paciente 4, pode-se observar uma estrutura semelhante a um vórtice *hairpin* , como visto para o paciente 1. Nesses pacientes, nota-se que o fluxo permaneceu próximo à parede aórtica durante o período sistólico.

Para todos os pacientes cujo aneurisma não cresceu ( Figura 6 ), estruturas semelhantes podem ser vistas em ambos os anos, ou seja, estruturas coerentes em formato toroidal estão presentes ao redor do jato de entrada, descolado da parede aórtica. Observe que o paciente 9 (grande *θ*_*I*_ ) apresenta uma estrutura toroidal próxima à raiz da aorta e uma estrutura grande e compacta aproximando-se da parede distal, que aumentou em tamanho no segundo ano.

Examinando a helicidade normalizada nas Figuras 5 e 6 , é possível observar, para todos os casos, regiões com valores absolutos acima de 0,6 (ou mesmo 0,8) e isso é consequência do fato de todas as aortas avaliadas apresentarem regiões de remodelamento vascular. Porém, um exame mais atento desses resultados mostra estruturas complexas para o grupo com crescimento do aneurisma, com vórtices contra-rotativos indicados pelo sinal oposto da helicidade. Para os pacientes que não apresentaram crescimento do aneurisma, a helicidade tem predominantemente um sinal, indicando apenas um sentido de rotação.

Comparando as estruturas coerentes e helicoidais de ambos os grupos, não há uma diferença clara entre eles, embora seja possível observar estruturas mais complexas no escoamento do grupo em que o aneurisma cresceu. Para esse grupo, as estruturas verticais são alongadas, terminando por formar um laço, com forte vórtice contra-rotativo, enquanto para o segundo grupo, o vórtice toroidal permanece próximo à raiz aórtica, girando predominantemente em uma direção. O vórtice mais intenso pode levar a diferentes níveis de estresse mecânico no endotélio aórtico.

**Figura 7:**
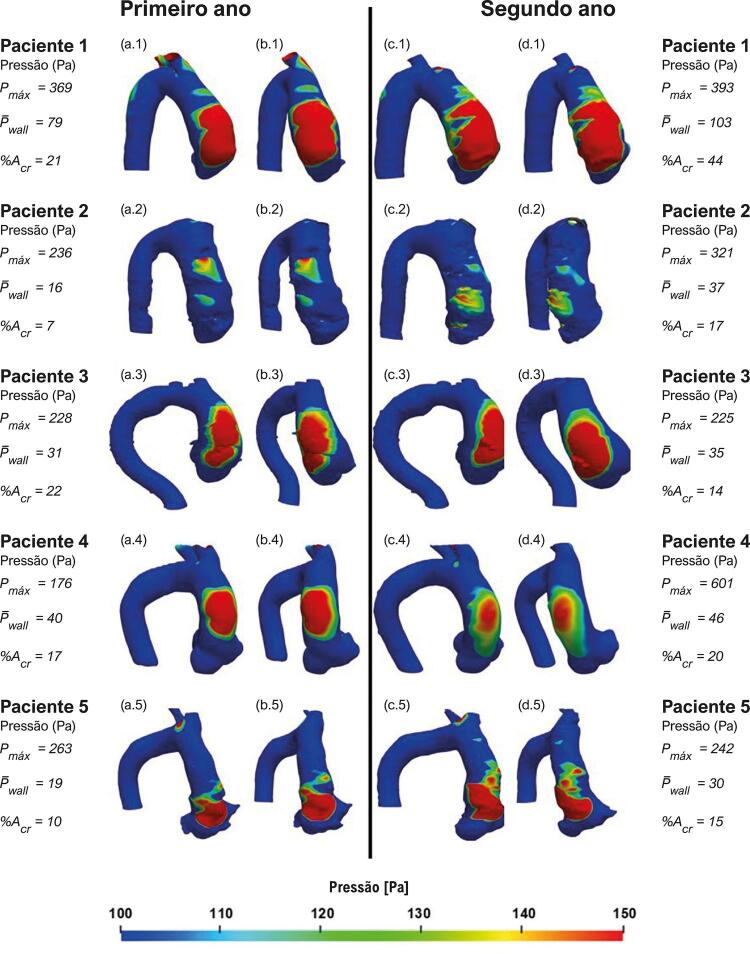
– Pressão na parede. Pacientes com crescimento de aneurisma: vista frontal (a) e vista lateral (b) do primeiro ano; vista frontal (c) e vista lateral (d) do segundo ano. Pressão máxima (P_max_) e pressão média (P_wall_) na região ascendente da aorta, percentual de área da aorta ascendente com pressão acima de 100 Pa (%A_er_).

As Figuras 7 e 8 ilustram, para ambos os anos, a distribuição de pressão na parede aórtica para os pacientes do grupo de aneurismas que cresceram e do grupo de aneurismas que não cresceram, respectivamente. Nessas figuras, apenas pressões acima de 100 Pa em relação à pressão de entrada são apresentadas, para ilustrar a região crítica na parede aórtica, ou seja, a região com altas pressões. Nessas figuras, também estão incluídas a pressão máxima e média da parede aórtica (i.e., *P*_*máx*_ e ***¯*** *P*_*wall*_ ), bem como a porcentagem da área da parede aórtica ascendente com alta pressão ( *%A*_*cr*_ ). Analisando essas figuras, pode-se associar ao jato de entrada, mostrado nas Figuras 3 e 4 , com sua posição de impacto na aorta, correspondendo à região do pico de pressão na parede aórtica anterior. Pode-se observar também, sua amplificação no segundo ano em 4 dos 5 pacientes no grupo de aneurisma com crescimento. Curiosamente, o paciente 4 apresenta o maior aumento na pressão máxima entre o primeiro e o segundo ano, atingindo valores acima de 600 Pa, porém com redução no tamanho da área de alta pressão, indicando concentração de alta pressão na região de impacto do jato.

**Figura 8:**
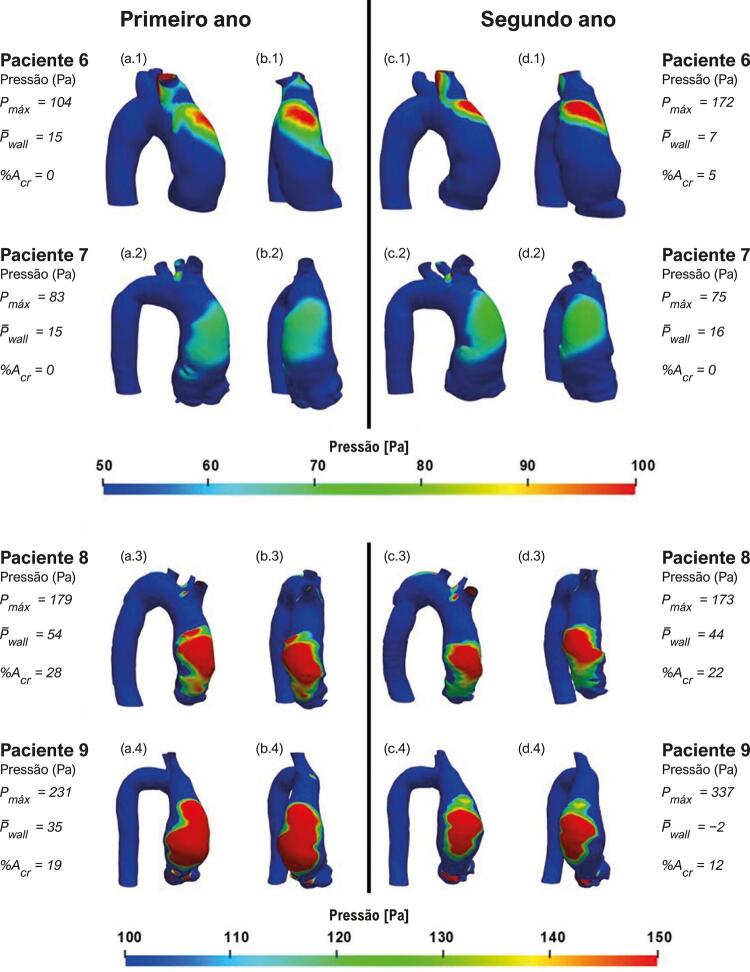
– Pressão na parede. Pacientes com crescimento de aneurisma: vista frontal (a) e vista lateral (b) do primeiro ano; vista frontal (c) e vista lateral (d) do segundo ano. Pressão máxima (P_max_) e pressão média (P_wall_) na região ascendente da aorta, (%A_er_) percentual de área da aorta ascendente com pressão acima de 100 Pa.

Notavelmente, há uma tendência para áreas maiores de pressões aumentadas no grupo de aneurisma com crescimento, com pressões máximas elevadas acima de 200 Pa, em comparação com o grupo de aneurisma sem crescimento, que demonstrou pressões máximas mais baixas (abaixo de 200 Pa) (com exceção do paciente 9 no grupo do aneurisma sem crescimento, que apresentou redução significativa da pressão média, o que pode explicar porque o aneurisma não cresceu). Além disso, houve um aumento na pressão média da parede para o grupo de aneurisma em crescimento e uma redução para o outro grupo, exceto para o paciente 7, cuja pressão máxima é bastante baixa, o que também poderia explicar porque seu aneurisma não cresceu.

### Perfil clínico

O perfil clínico da população estudada é mostrado na Tabela 2 . Dados clínicos de dois pacientes não se encontravam disponíveis, sendo excluídos da tabela. Nesses dois casos, paciente do sexo feminino de 60 anos de idade e outro do sexo masculino de 70 anos, o aneurisma cresceu. Todos os sete pacientes eram hipertensos. A maioria tinha dislipidemia, tabagismo atual ou passado, fração de ejeção ventricular esquerda preservada e níveis controlados de pressão arterial.

**Tabela 2:** – Perfil clínico da população

	População* (n=7)	Aneurisma cresceu (n=3)	Aneurisma não cresceu (n=4)
**Idade** (anos ± DP)	65,1 ± 8,1	66 ± 8	64,5 ± 8,2
**Sexo, masculino (n, %)**	5 (71,4)	3 (42,9)	2 (28,6)
**Hipertensão** (n, %)	7 (100)	3 (42,9)	4 (57,1)
**Diabetes** (n, %)	3 (42,9)	1 (14,3)	2 (28,6)
**Dislipidemia** (n, %)	5 (71,4)	2 (28,6)	3 (42,9)
**Doença renal crônica** (n, %)	2 (28,6)	1 (14,3)	1 (14,3)
F **ibrilação atrial** (n, %)	2 (28,6)	1 (14,3)	1 (14,3)
**Doença neurológica** (n, %)	1 (14,3)	0 (0)	1 (14,3)
**Tabagismo atual ou passado** (n, %)	4 (57,1)	2 (28,6)	2 (28,6)
**Doença aterosclerótica obstrutiva coronariana** (n, %)	1 (14,3)	1 (14,3)	0 (0)
**Doença cerebrovascular** (n, %)	1 (14,3)	0 (0)	1 (14,3)
**Classe funcional I e II NYHA** (n, %)	6 (85,7)	3 (42,9)	3 (42,9)
**Angina** (n, %)	2 (28,6)	1 (14,3)	1 (14,3)
**Síncope ou lipotimia** (n, %)	0 (0)	0 (0)	0 (0)
**Índice de massa corporal** (kg/m^2^ ± DP)	29,5 ± 2	28,3 ± 2,1	30,3 ± 1,8
**Fração de ejeção do ventrículo esquerdo** (Teicholz % ± DP)	62,1 ± 8,7	56,6 ± 9,8	66,2 ± 4,7
**Pressão arterial sistólica** ≤ 130 mmHg (n, %)	5 (71,4)	2 (28,6)	3 (42,9)
**Pressão arterial diastólica** ≤ 85 mmHg (n, %)	6 (85,7)	3 (42,9)	3 (42,9)
**Frequência cardíaca** < 70 bpm (n, %)	5 (71,4)	2 (28,6)	3 (42,9)

Perfil clínico de 7 pacientes da população* (dados clínicos de 2 pacientes não estavam disponíveis); DP: desvio padrão; NYHA: classe funcional I e II de New York Heart Association significa sem limitações ou com sintomas leves e poucas limitações nas atividades cotidianas; bpm: batimentos por minuto.

## Discussão

A simulação computacional hemodinâmica tem se mostrado uma promissora área da pesquisa científica translacional^15^ com possível aumento de aplicações clínicas em um futuro próximo. No presente estudo, pacientes foram classificados como portadores de AAAs com crescimento e sem crescimento de acordo com o volume da região de interesse. Raghavan et al.^30^ demonstraram uma melhor correlação da possível ruptura do aneurisma com o volume da aorta ascendente e alto estresse na parede, do que apenas com o diâmetro da aorta ascendente.

Como mostra a Tabela 1 , todos os pacientes identificados como tendo crescimento do aneurisma apresentaram grandes *θ*_*I*_ e pequenos *θ*_*II*_ ( Figura 2c ). Por outro lado, entre aqueles sem crescimento de aneurisma ( Tabela 1 ), nenhuma tendência foi notada. Pode-se sugerir uma correlação do crescimento do aneurisma com o ângulo entre a entrada da geometria aórtica e o tronco braquiocefálico, uma vez observado que pacientes com angulações maiores que 94° apresentaram crescimento do aneurisma ao longo dos anos. Devido à forma da aorta, o jato de entrada colide fortemente com a parede anterior, elevando os níveis de pressão e tensão cisalhante, induzindo à formação de estruturas semelhantes a *hairpin* e com forte recirculação. Esses fenômenos foram observados no grupo que apresentou crescimento do aneurisma da aorta, o que poderia, por conseguinte, contribuir com o processo de remodelamento aórtico.

*θ*_*I*_ e *θ*_*II*_ ( Figura 2c ), listados na Tabela 1 , correspondentes a cada grupo, foram mensurados independentemente por dois observadores, consoante com o descrito na seção “Métodos”. Baseado em um método de mensuração manual de ângulos, o CCI entre os dois observadores independentes foi 0,99, o que foi considerado excelente,^28^ demostrando alta reprodutibilidade do método. Nenhuma diferença estatística foi encontrada em ambos os ângulos na relação dos grupos com e sem crescimento do aneurisma, e uma amostra de nove indivíduos não permitiu maiores inferências estatísticas. No entanto, a alta taxa de concordância entre os observadores pode permitir, com um maior número de pacientes analisados, encontrar associações estatísticas significativas em um futuro próximo. Como um estudo sem precedentes, a presente pesquisa pode ser considerada uma prova de conceito em termos de pesquisa numérica e um estudo de tecnologia translacional disruptiva.

Comparando o campo de escoamento dentro da aorta dos nove pacientes, com um intervalo de tempo de pelo menos um ano, diferentes estruturas coerentes foram observadas. Estruturas toroidais com baixas velocidades descoladas da parede da aorta distal foram identificadas entre os pacientes que não apresentaram crescimento do aneurisma. Entre aqueles com crescimento do aneurisma, dois tipos foram notados: estruturas como hairpin e estruturas toroidais com altas velocidades.

Uma vez que a distribuição da helicidade indica duas direções de rotação nos pacientes cujos aneurismas cresceram, é possível inferir que nesses casos o escoamento é provavelmente mais perturbado. Outra possibilidade é que o fluxo dentro de um aneurisma aórtico contenha regiões de forte recirculação, que são responsáveis pela formação de estruturas coerentes presentes ao longo da aorta. Michel et al.^37^ discutiram que alterações nos padrões de fluxo sanguíneo podem induzir o desenvolvimento aneurismático na curvatura externa da aorta ascendente, devido à alta tensão cisalhante na parede transversal e impedância mecânica no lado convexo.

Biasetti et al.^38^ correlacionaram a presença de vórtices com alta tensão cisalhante em pacientes com aneurismas de aorta abdominal. No presente estudo, estruturas coerentes verticais também podem ser vistas próximo à parede distal da aorta, evoluindo, em alguns casos, para estruturas semelhantes a *hairpin* . Essas diferenças na estrutura do fluxo de pacientes nos dois diferentes grupos (com e sem crescimento do aneurisma) podem indicar a possibilidade de um processo de remodelamento do aneurisma da aorta.

Uma hipótese para explicar esses achados é a transferência de estresse à adventícia por perda de deposição de elastina e colágeno, promovendo contínua adaptação por causa da distribuição da tensão e, consequentemente, mudanças na forma do AAAs.^39^

Existem algumas limitações a esta pesquisa. Primeiramente, neste estudo, a varredura por ATC foi realizada por diferentes equipes médicas. Como resultado, a qualidade dos exames não foi a mesma, o que pode ter afetado a acurácia da comparação das imagens e a definição da forma da aorta. Em segundo lugar, a pequena população deste estudo limita qualquer correlação entre padrões clínicos e crescimento do AAAs. Em terceiro lugar, dados clínicos de dois pacientes não estavam disponíveis, assim, eles não foram incluídos na Tabela 2 . Além disso, a presente análise foi realizada considerando-se a situação mais crítica durante a sístole, correspondendo à vazão máxima em um ciclo cardíaco, permitindo considerar a aorta como estrutura rígida e estática no momento do pico sistólico.

Claramente, há muitos fatores que precisam ser considerados e que interferem no prognóstico dos pacientes em relação ao crescimento do aneurisma da aorta, como sua idade, comorbidades e uso de medicamentos. O ângulo entre a entrada da aorta e o tronco braquiocefálico pode ser uma importante variável a ser considerada, assim como a estrutura do escoamento e a distribuição da pressão na parede anterior da aorta, que pode ser numericamente determinada.

A partir da distribuição de pressão na parede da aorta, é possível observar que a pressão média aumentou mais de 13% para todos os pacientes que tiveram crescimento do aneurisma, enquanto, para aqueles sem crescimento patológico, a pressão média decresceu mais de 18% para todos os pacientes, com exceção de um. Esse resultado indica que essa variável pode influenciar no crescimento do AAAs.

Estudos futuros com maior número de pacientes e maior tempo de acompanhamento são necessários para definir a relação entre padrão de fluxo sanguíneo e processo de remodelamento aórtico. A simulação numérica é uma ferramenta não invasiva que pode ser utilizada juntamente com a varredura de ATC para estimar a tendência de crescimento do AAAs. A identificação de diferentes perfis de risco entre pacientes com AAAs pode abrir caminho para avaliação médica diferenciada para esses pacientes. Todo o exposto pode levar a um avanço na medicina personalizada e ajudar a decifrar a fisiopatologia dessa grave doença.

Considerando que algumas características do escoamento podem estar associadas ao crescimento do aneurisma, o estudo de padrões de fluxo na aorta ascendente pode ajudar a predizer o processo de remodelamento de AAAs.

## Conclusões

Este estudo piloto mostrou que a CFD aplicada em ATC pode, em um futuro próximo, ser uma ferramenta para ajudar a identificar padrões de comportamento do escoamento associados com o processo de remodelamento de AAAs. Vórtices foram formados na região posterior do jato incidente na parede aórtica, gerando estruturas complexas no grupo com crescimento do AAAs. Como consequência, houve um aumento da pressão média na parede anterior da aorta no grupo que apresentou crescimento do aneurisma, enquanto, para os casos sem crescimento do aneurisma, essa variável decresceu.
