# Supplementary material for: Fluidodinâmica Computacional na Avaliação do Risco Futuro de Aneurismas de Aorta Ascendente
Source: Arq Bras Cardiol. 2022 Feb 14;118(2):448–60. [Article in Portuguese] doi: 10.36660/abc.20200926 (PMC8856676; doi:10.36660/abc.20200926)
Supplement: Translation

# Translation: Computational Fluid Dynamics to Assess the Future Risk of Ascending Aortic Aneurysms

**Authors:** Gabriela de C. Almeida, Bruno Alvares de Azevedo Gomes, Fabiula Schwartz de Azevedo, Karim Kalaun, Ivan Ibanez, Pedro S. Teixeira, Ilan Gottlieb, Marcelo M. Melo, Glaucia Maria Moraes de Oliveira, Angela O. Nieckele

**Affiliations:** 1 Brazil Departamento de Engenharia Mecânica, Pontifícia Universidade Católica do Rio de Janeiro, Rio de Janeiro, RJ – Brazil; 2 Brazil Departamento de Cardiologia, Universidade Federal do Rio de Janeiro, Rio de Janeiro, RJ – Brazil; 3 Brazil FIT Center – Clínica de Performance Humana, Niterói, RJ – Brazil; 4 Brazil Casa de Saúde São José, Rio de Janeiro, RJ – Brazil; 5 Brazil Instituto Nacional de Cardiologia, Rio de Janeiro, RJ – Brazil

**Keywords:** Hemodynamics, Aortic Aneurysm, Thoracic, Patient-Specific Modeling

## Abstract

**Background:**

A methodology to identify patients with ascending aortic aneurysm (AsAA) under high risk for aortic growth is not completely defined

**Objetive:**

This research seeks to numerically characterize the aortic blood flow by relating the resulting mechanical stress distribution with AsAA growth.

**Methods:**

Analytical, observational, single-center study in which a computational fluid dynamics (CFD) protocol was applied to aortic computed tomography angiogram (CTA) images of patients with AsAA. Two CTA exams taken at a minimum interval of one year were obtained. From the CTA-gathered images, three-dimensional models were built, and clinical data were registered. Study of velocity field and coherent structures (vortices) was performed aiming to relate them to the presence or absence of aneurysm growth, as well as comparing them to the patients’ clinical data. The Kolmogorov-Smirnov test was used to evaluate the normality of the distribution, and the non-parametric Wilcoxon signed-rank test, for non-normal distribution, was used to compare the paired data of the aortic angles. Statistical significance was set at 5%.

**Results:**

The incident jet in the aortic wall generated recirculation areas in the posterior region of the jet, inducing complex vortices formation in the group with aneurysm growth, leading to an average pressure increase in the ascending aortic wall between exams. In the group without aneurysm growth, the average pressure decreased.

**Conclusion:**

This pilot study showed that CFD based on CTA may in the near future be a tool to help identify flow patterns associated with AsAA remodeling process.

## Introduction

Ascending aortic aneurysm (AsAA) is usually asymptomatic and progresses imperceptibly.^1 , 2^ However, its complications, such as rupture and dissection, are catastrophic events.^3^ Some aneurysms do not grow, while others increase significantly in size in a short period. The variables related to the AsAA growth are not known precisely.^2 , 4^

The AsAA formation is a multifactorial degenerative process, resulting from hemodynamic factors and biological processes.^5^ According to Hope et al.,^4^ changes in the flow pattern along the ascending aorta are related to the remodeling process, and, thus, may influence aneurysm growth.

Computational Fluid Dynamics (CFD) has gained great interest as a complementary tool to improve the understanding of cardiovascular disease pathogenesis and progression, in addition to proving itself suitable for minimally invasive assessments.^6 , 7^ As highlighted by Morris et al.^7^ CFD combined with population-scale numerical models have the potential to reduce the risks, costs, and time associated with clinical trials.

Through CFD studies of aortic flow, regions with high rates of wall shear stress and pressure can be identified,^8^ suggesting an association of these variables with aneurysmal dilatation.^9^ These observations were discussed by Gülan et al.,^10^ who found large rotational regions formed during the systolic phase, showing qualitative similarity with the vortex ring formation, i.e., a central jet surrounded by two large vortices. From the fluid mechanics viewpoint, the flow through an abrupt expansion, such as that between the aortic annulus and the dilated ascending portion, leads to separation of the boundary layer and formation of a separation zone. Hence, there might be a correlation between flow separation, turbulence, pressure loss, and aneurysm growth.^10^

In addition, the presence of coherent structures inside the aorta can be applied to explain the formation of high shear stress and pressure regions, which can contribute to the aortic remodeling process.^11^Figure 1 shows the ascending aorta region of interest with the main flow variables, which is the central illustration of the present study. The goal of this pilot study is to investigate the blood flow behavior along the ascending aorta aiming to identify some flow patterns that might be associated with AsAA growth.

**Figure 1:** – Main area of interest of the ascending aorta, with main flow variables.

## Methods

### Study guidelines

The present work is an analytical, observational, single-center study, in which a CFD protocol was applied to aortic computed tomography angiogram (CTA) images. This study was registered in the National Research Ethics Committee of the Health Ministry and approved by the local Research Ethics Committee (CAAE 86716318.3.0000.5272, Number: 2.750.919). All participants provided written informed consent in accordance with Resolution 466/2012 of the National Health Board.

### Patients

This study assessed nine patients with AsAA selected from 100 consecutive patients from the aortic disease outpatient clinic of a tertiary health center. Patients with AsAA diagnosis were included, and all of them had stable disease. Patients with collagen pathologies and previous aortic and cardiac surgery were excluded. All patients had two available images of CTA scan with a minimum interval of one-year between the exams. The images were obtained due to clinical indications for follow-up, not specifically for the present study. Patients were under medical treatment according to recent guidelines on AsAA.^12^

### Geometry: segmentation and the three-dimensional model

The aorta geometry was built based on a CTA scan obtained with a 64-slice scanner SOMATOM Sensation 64 (Siemens, Germany). The selected CTA slices were spanned from the aortic annulus to the thoracic aorta. Information, such as pixel size and distance slice, was obtained to further adjust the three-dimensional (3D) aortic model to its actual size. The image segmentation was performed with the software FIJI, an open-source image processing software based on ImageJ, focused on biological-image analysis.^13^

Two 3D aortic models for each patient were super-imposed to provide a spatial reference for comparison between the exams, aiming to overlap the images of the beginning of the brachiocephalic trunk and the right coronary arteries ( Figure 2a ). The aorta corresponding to the first year is gray, and the one corresponding to the second-year exam is blue. Once the arteries were overlapping, the aortic valve and the descending part were sectioned ( Figure 2a ), ensuring that the inflow and outflow sections had the same spatial reference. Figure 2b shows the study’s main area of interest, which is the ascending aorta. The entry flow plane was positioned in the centroid of the aortic annulus, so that the x-axis crosses the centroid of the left main coronary artery, pointing towards the anterior aortic wall, and the y-axis pointing to the right coronary artery.

**Figure 2:** – (a) Comparison criteria between aortas of the same patient in different years. The yellow arrow indicates superposition of the brachiocephalic trunk of the aortas, with the dotted line indicating its beginning, and overlapping of the right coronary arteries, with the aortic valve and descending aorta portion sectioned; (b) the main area of interest of the aortas; (c) Angle I (θ_I_) and Angle II (θ_II_); (d) inlet and outlet conditions.

Since the flow field depends directly on the aortic shape, two angles ( *θ*_*I*_ and *θ*_*II*_ ) were defined between the inflow plane and the brachiocephalic trunk outlet, aiming to relate it with the flow pattern that can induce aneurysm growth ( Figure 2c ). *θ*_*I*_ was defined as the angle between the line connecting the brachiocephalic trunk centroid with the left main coronary artery centroid and the x-axis centered in the inflow plane. *θ*_*II*_ was formed by the angle between the line that connects the brachiocephalic trunk centroid with a point on the extreme position on the x-axis of the inflow plane, and the line connecting this point with the extreme position on the y-axis of the inflow plane. Figure 2d illustrates the valve inlet and the four outlet sections.

### Flow modeling

To examine the correspondence of the flow field with the patient’s pathology, we focused on the ascending aortic portion during ventricular systole, when the aortic walls are distended and provide maximum diameter. During systolic peak, the aortic diameter is maximum and compliance is small, allowing considering the aortic wall stiff.^14^ In addition, the present analysis was performed considering the most critical situation during the systole, corresponding to the maximum physiological flow rate, *Q*_*in*_ = 25 l/min,^15^ flowing in time-averaged steady turbulent manner.^16^ To model the turbulence, the time averaged two-equation κ−ω SST model^17^ was selected based on the recommendation by Celis et al.^18^ , who performed a numerical comparison with the experimental data by Gomes et al.^19^ on similar aortic anatomy. The effects of gravity were also neglected since the pressure variations are dominant over the force of gravity.

Blood was modeled as a Newtonian fluid since high shear rates^20^ are found in patients with ascending aortic aneurysm (deformation rate above 50 s^-1^).^21^ In addition, under normal conditions, blood can be considered an incompressible fluid.^22^ Density was set at *ρ* = 1054 kg/m^3^The dynamic viscosity was defined as *μ* = 7.2 cP to allow a direct comparison with in vitro experimental data.^18 , 19 , 23^ Additionally, a few tests were performed and the results obtained with viscosities equal to 3.5 cP and 7.2 cP provided equivalent results, with pressure differences inferior to 0.01 mm Hg in the region of interest of the ascending aorta. These tests corroborate the findings by Becsek et al.,^24^ who evaluated viscosities equal to 4 cP, 6 cP, and 8 cP and showed negligible effect on the mean flow field and a very small effect on the location of the turbulent breakdown.

For all cases, the same outlet flow-rate distributions (inflow percentage) were imposed based on average values in the human body, according to Alastruey et al.,^15^ as shown in Figure 2d : descending aorta, 69.1%; brachiocephalic trunk, 19.3%; left carotid artery, 5.2%; and left subclavian artery, 6.4%.

### Numerical simulation

The present analyses were performed using ANSYS Fluent v18.1 software, which solves the conservation equations discretized based on the finite volume method.^25^ Simulations were post-processed with the ANSYS CFD-Post tool^26^ and with the open-source Paraview.^27^

The discretized equations were obtained with the “Power Law scheme”.^25^ The pressure-velocity coupling was handled with the SIMPLE algorithm,^25^ and the maximum convergence residual error was set as 10^-6^for all equations.

A mesh with 400 000 nodes was applied to all cases studied, which were determined based on a mesh independent test, by guaranteeing a pressure drop variation smaller than 0.3% at the main area of interest (ascending aorta), when the mesh size was doubled.

### Statistical analysis

The anatomic shape of each patient’s aorta can be inferred by the reference angles *θ*_*I*_ and *θ*_*II*_ ( Figure 2c ), which were measured by two observers independently. Continuous variables with normal distribution were presented as mean and standard deviation. Continuous variables without normal distribution were presented as median and interquartile range. Inter-observer reproducibility was calculated by use of the intraclass correlation coefficient (ICC) for *θ*_*I*_ and *θ*_*II*_ measurements. The following criteria for reliability^28^ was applied: poor (ICC ≤ 0.50); moderate (0.50 < ICC ≤ 0.75); good (0.75 < ICC ≤ 0.90); excellent (ICC > 0.90).

The Kolmogorov-Smirnov normality test was used to examine if variables had a normal distribution. Due to non-normal distribution, the Wilcoxon signed-rank test, a non-parametric test, was applied for comparison of paired data (dependent samples) between the *θ*_*I*_ and *θ*_*II*_ measurements. Statistical significance was set at 5%. All statistical analyses were conducted using IBM SPSS software, version 26.^29^

## Results

### Aortic shape and angles

Patients were divided into two groups based on the area of interest volume.^30^ The aneurysm was considered as having grown when the difference between the volume of the area of interest increased by 10% or more, comparing the two CTAs for each patient. Table 1 presents the percentage of the volume difference and the time interval between the CTA scans of each patient. There was no statistical difference between the measurements of two independent observers. The resulting ICC was 0.99, considered excellent.

**Table 1:** – Patient classification due to aneurysm growth

	Patient	Volume variation (%)	Interval between scans (years)	Sex	Age (years)	Year	Angle 1 (º)	Angle 2 (º)
**Aneurysm growth**	1	18.0	2	M	77	1	138.80	63.56
						2	144.85	56.61
	2	10.0	1	F	60	1	116.64	51.34
						2	94.74	42.69
	3	15.6	2	M	70	1	116.51	42.61
						2	126.91	46.07
	4	10.5	3	M	63	1	124.14	51.71
						2	128.03	51.59
	5	12.5	2	M	58	1	135.79	51.30
						2	134.74	67.86
**No aneurysm growth**	6	0.50	1	F	63	1	94.90	78.84
						2	88.33	91.39
	7	-4.50	2	F	52	1	77.19	70.86
						2	94.20	71.99
	8	-0.13	2	M	74	1	119.22	64.07
						2	121.53	56.89
	9	-0.20	3	M	69	1	136.41	66.14
						2	137.09	58.99

*M: male; F: female.*

As shown in Table 1 , the volume variation of patients 6, 8, and 9 is negligible. Based on the difference between the aortic volume measurements, a volume variation equal or superior to 10% (patients 1 to 5) was considered as aneurysm growth, while a volume variation below that threshold value (patients 6 to 9) was considered as no aneurysm growth. The median and interquartile range of the volume variation in patients with aneurysm growth were 12.5 [10.25 – 16.8]%; without "aneurysm growth, (-0.16) [(-2.35) –(0.18)] %; and in the total population, 10 [(-0.16) –( 14.05)] %."

Table 1 shows the measurements corresponding to the first and second years for all patients. All patients identified as having aneurysm growth presented large Angle I ( *θ*_*I*_ *≥* 94^o^) and small Angle II (51^o^ *≤ θ*_*II*_ *≤* 68^o^). However, when examining the angles corresponding to the patients without aneurysm growth, a clear tendency was not observed. There were patients with large and small *θ*_*I*_ and *θ*_*II*_ . Patients 6 and 7 clearly presented a different range of angles, but the same was not true for patients 8 and 9. The *θ*_*I*_ measurement in patients with aneurysm growth was 127.47 [116.64 – 135.79]°; without aneurysm growth, 119.22 [91.26 – 128.97]°; and in total population, 121.53 [94.9 – 135.79]°. The *θ*_*II*_ measurement in patients with aneurysm growth was 51.46 [46.07 – 56.61]°; without aneurysm growth, 68.5 [61.53 – 75.41]°; and in the total population, 57.94 [51.34 – 67.86]°.

### Flow characteristics

To visualize the flow field inside the aorta, using frontal and lateral aortic views, a grey color axial velocity iso-surface ( *w* ) equal to 50% of inlet axial velocity ( *w*_*in*_ ) was drawn ( Figure 3 and Figure 4 correspond to patients with and without aneurysm growth, respectively). This variable allowed for the identification of the shape of the inlet jet. Velocity streamlines were also included in the figures to aid in the identification of flow recirculation. To evaluate the turbulence level, those lines were colored according to the turbulent kinetic energy (κ). For this analysis, two angles of visualization were defined, corresponding to the frontal and lateral views of the aorta. Analyzing Figure 3 , for all five patients with aneurysm growth, the inlet jet is directed towards the anterior aortic wall. In addition, large rotational regions can be seen, except for patient 4, who, as will be shown later, had a substantial increase in the pressure level at the ascending aortic wall. These results corroborated remarks by Gülan et al.^10^ , who suggested that the presence of rotational regions might be linked with aneurysm growth. For patients 6 and 7, whose aneurysms did not grow ( Figure 4 ), since *θ*_*I*_ ( Table 1 ) is smaller, the resulting flow field extends through the aorta, since the jet does not impinge on the vessel wall. Although longer inlet jets can also be observed for patients 8 and 9 concerning patients 1 to 5, due to their large *θ*_*I*_ , the inlet jet also impinges on the anterior wall, but no recirculation region is observed, i.e., the streamlines follow a smooth path along the aorta. Note that the flow field of patient 6, whose aneurysm did not grow, shows recirculation regions, but it is located further up in the ascending region. With regards to the turbulence level, no significant differences were observed for both groups. For all cases, 5% turbulence intensity was imposed at the valve inlet and, for both groups, an average turbulence intensity between 1% and 2% was obtained.

**Figure 3:** – Axial-velocity iso-surface (w/w_in_= 0.5) and streamlines colored according to the turbulent kinetic energy scale. Patients with aneurysm growth: (a) frontal and (b) lateral view of the first year; (c) frontal and (d) lateral view of the second year.

**Figure 4:** – Axial-velocity iso-surface (w/w_in_= 0.5) and streamlines colored according to the turbulent kinetic energy scale. Patients without aneurysm growth: (a) frontal and (b) lateral view of the first year; (c) frontal and (d) lateral view of the second year.

According to Weigang et al.^31^ some cases with aneurysm growth present the formation of large rotational regions, with a vortex ring surrounding a central jet with two large vortices. Thus, to identify coherent flow structures, we employed the *Q* -criteria,^32^ defined as


$$Q=\left(\Omega_{ij}\Omega_{ij}-S_{ij}S_{ij};S_{ij}=\frac{1}{2}\left(\frac{\partial u_{i}}{\partial x_{j}}+\frac{\partial u_{j}}{\partial x_{i}}\right);\Omega_{ij}=\frac{1}{2}\left(\frac{\partial u_{i}}{\partial x_{j}}-\frac{\partial u_{j}}{\partial x_{i}}\right)\right.$$ (1)

where a positive *Q* means domination of the vorticity magnitude *Ω* over the strain rate *S* . Further, *Q* is related to regions of low pressure, which can also be associated with the presence of a coherent structure. Additionally, the normalized helicity^33^


$$H=\xi_{i}u_{i}/\left[\sqrt{\xi_{k}\xi_{k}}\sqrt{u_{\ell }u_{\ell }}\right];\xi_{i}=\epsilon_{ijk}\partial u_{j}/\partial u_{i}$$ (2)

is also examined, where *ξ*_*i*_ is the vorticity, and *ϵ*_*i* *jk*_ is the Levi-Civita operator. Helicity assesses the flow propensity to form coherent vortices, representing the amount of linkage of flow vortex lines.^34^ According to Garcia et al.,^35^ helical flow might promote aortic dilatation, therefore normalized helicity might aid in the characterization of aortic and heart valve diseases. In addition, when the directions of the vorticity and velocity vector are the same, normalized helicity is *H = 1* ; when they are opposite, *H = –1* and for orthogonal vectors *H = 0* . Thus, helicity is a quantitative indication of the helical flow around the principal flow direction in the aortic region.^35^ Helicity values of 1 or −1 represent the vortex core, which is characterized by strong vortex roll-up. Gallo et al.^36^ have indicated that an absolute normalized helicity value higher than 0.6 represents the possibility of arterial remodeling, but Garcia et al.^35^ have indicated a different threshold of 0.8.

A *Q* iso-surface for each patient, normalized by the inlet velocity and effective diameter, is plotted in Figure 5 and Figure 6 , corresponding to the two groups. Each *Q* iso-surface was colored with the dimensionless helicity *H* , on a scale represented on the bottom of the figures.

**Figure 5:** – Q iso-surface colored according to normalized helicity scale. Patients with aneurysm growth: (a) first year; (b) second year.

**Figure 6:** – Q iso-surface colored according to normalized helicity scale. Patients without aneurysm growth: (a) first year; (b) second year.

Analyzing Figure 5 for patient 1, who presented the largest aortic dilatation, a large vortex structure similar in shape to a hairpin vortex can be seen reaching the distal wall, in both years. The angular curvature of patient 1 may have caused the formation of a detached vortex. Patient 2 (smallest dilatation of the group) presented a toroidal vortex near the aortic root, with a longitudinal vortex along the distal wall. The same phenomenon occurs for patients 3 and 5. Examining the Q iso-surface for patient 4, a hairpin-like structure can be seen close to the wall, as seen for patient 1. Those patients showed that the flow remained attached to the aortic walls during the systolic period.

For all patients whose aneurysm did not grow ( Figure 6 ), similar structures can be seen for both years, i.e., coherent structures in a toroidal format are present around the inlet jet, detached from the aortic wall. Note that patient 9 (large Angle I) presents a toroidal structure near the aortic root and a large, compact structure approaching the distal wall, which increased in size in the second year.

Examining the dimensionless helicity in Figure 5 and Figure 6 , regions with absolute normalized helicity values above 0.6 (or even 0.8) can be seen in all cases, and this is consequent to the fact that all evaluated aortas present vascular remodeling regions. However, a closer look into these results shows more complex structures for the group with aneurysm growth, with counter-rotating vortex indicated by the opposite sign of the helicity. For the patients without aneurysm growth, the helicity has predominantly one sign, indicating only one direction of rotation.

Comparing the coherent structures and helicity of both groups, there is not a clear difference between them, although, a slightly more complex flow structure was observed for the group with aneurysm growth. For this group, vertical structures are stretched, ending up forming a loop, with a strong counter-rotating vortex, while, for the second group, the toroidal vortex remains close to the aortic root, predominantly rotating in one direction. The stronger vortex might lead to different stress levels at the aortic endothelium.

Figure 7 and Figure 8 illustrate the pressure distribution at the aortic wall for patients with and without aneurysm growth, respectively, for both years. In these figures, only pressures above 100 Pa in relation to the inlet pressure are shown, to illustrate the critical region at the aortic wall, i.e., the region under high pressures. In these figures, the maximum and average aortic wall pressure (i.e., *P*_*máx*_ and ***¯*** *P*_*wall*_ ) were also included, as well as the percent area of the ascending aorta under high pressure ( *%A*_*cr*_ ). Analyzing these figures, one can associate the inlet jet, shown in Figure 3 and Figure 4 , with its impingement position, corresponding to the pressure peak region at the anterior aortic wall. One can also observe its amplification in the second year in 4 out of 5 patients in the growing aneurysm group. Interestingly, patient 4 has the largest increase in maximal pressure between the first and second years, reaching values > 600 Pa, however with a reduction in the size of the high-pressure area, indicating high-pressure concentration in the impingement region.

**Figure 7:** – Wall pressure. Patients with aneurysm growth: (a) frontal and (b) lateral view of first year; (c) frontal and (d) lateral view of second year. Maximum pressure (P_max_) and average wall pressure (¯P_wall_) in the ascending aortic region, and percent area of the ascending aorta with pressure above 100 Pa (%A_er_).

**Figure 8:** – Wall pressure. Patients without aneurysm growth: (a) frontal and (b) lateral view of first year; (c) frontal and (d) lateral view of second year. Maximum pressure (P_max_) and average wall pressure (¯P_wall_) in the ascending aortic region, and percent area of the ascending aorta with pressure above 100 Pa (%A_er_).

Notably, there is a tendency towards larger areas of increased pressures in the growing aneurysm group, with elevated maximum pressures above 200 Pa, compared to the non-growing aneurysm group, which demonstrated lower maximum pressures (below 200 Pa), except for patient 9 in the non-growing aneurysm group, who presented a significant reduction in the average pressure, which might explain the lack of aneurysm growth. Further, there was an increase in the average wall pressure in the growing aneurysm group and a reduction in the other group, except for patient 7, whose maximum pressure was quite low, which could also explain the lack of aneurysm growth.

### Clinical profile

The clinical profile of the population studied is shown in Table 2 . Clinical data of two patients were not available, so they were excluded from the table. In these two cases, a 60-year-old female and a 70-year-old male, there was aneurysm growth. All seven patients had high blood pressure. Most were current or ex-smokers, had dyslipidemia, preserved left ventricular ejection fraction, and desirable systolic and diastolic blood pressure levels.

**Table 2:** – Clinical profile of the population

	Population* (n=7)	Aneurysm growth (n=3)	No aneurysm growth (n=4)
**Age** (years ± SD)	65.1 ± 8.1	66 ± 8	64.5 ± 8.2
**Gender, male** (n, %)	5 (71.4)	3 (42.9)	2 (28.6)
**Hypertension** (n, %)	7 (100)	3 (42.9)	4 (57.1)
**Diabetes** (n, %)	3 (42.9)	1 (14.3)	2 (28.6)
**Dyslipidemia** (n, %)	5 (71.4)	2 (28.6)	3 (42.9)
**Chronic kidney disease** (n, %)	2 (28.6)	1 (14.3)	1 (14.3)
**Atrial fibrillation** (n, %)	2 (28.6)	1 (14.3)	1 (14.3)
**Neurological disease** (n, %)	1 (14.3)	0 (0)	1 (14.3)
**Current or past smoking** (n, %)	4 (57.1)	2 (28.6)	2 (28.6)
**Obstructive coronary atherosclerotic disease** (n, %)	1 (14.3)	1 (14.3)	0 (0)
**Cerebrovascular disease** (n, %)	1 (14.3)	0 (0)	1 (14.3)
**Functional class I and II NYHA** (n, %)	6 (85.7)	3 (42.9)	3 (42.9)
**Angina** (n, %)	2 (28.6)	1 (14.3)	1 (14.3)
**Syncope or lipothymia** (n, %)	0 (0)	0 (0)	0 (0)
**Body mass index** (kg/m^2^± SD)	29.5 ± 2	28.3 ± 2.1	30.3 ± 1.8
**Left ventricle ejection fraction** (Teicholz % ± SD)	62.1 ± 8.7	56.6 ± 9.8	66.2 ± 4.7
**Systolic blood pressure** ≤ 130 mm Hg (n, %)	5 (71.4)	2 (28.6)	3 (42.9)
**Diastolic blood pressure** ≤ 85 mm Hg (n, %)	6 (85.7)	3 (42.9)	3 (42.9)
**Heart rate** < 70 bpm (n, %)	5 (71.4)	2 (28.6)	3 (42.9)

Clinical profile of 7 patients of the population* (clinical data of 2 patients were not available); SD: standard deviation; NYHA: New York Heart Association functional class I and II means no limitation or mild symptoms with slight limitation in ordinary activity; bpm: beats per minute.

## Discussion

Hemodynamics simulation has been a promising area of translational scientific research^15^ for possible clinical applications in the near future. In this study, patients were classified as AsAA with growth or without growth according to the volume of the area of interest. Raghavan et al.^30^ have shown a better relationship of possible aneurysm rupture with the ascending aorta volume and high wall stresses, rather than the ascending aorta diameter.

As shown in Table 1 , all patients identified as having aneurysm growth presented large *θ*_*I*_ and small *θ*_*II*_ ( Figure 2c ). On the other hand, among patients without aneurysm growth ( Table 1 ), no tendency was noted. A correlation of aneurysm growth with the angle between the aortic inlet and the brachiocephalic trunk may be suggested, because for patients with angulation greater than 94°, aneurysm growth was present over the years. Due to the aortic shape, the incoming jet strongly impinges on the anterior wall, raising the pressure levels and shear stress, inducing the formation of hairpin-like structures with strong recirculation. These phenomena were observed in the group with aortic aneurysm growth, which might therefore contribute to the aortic remodeling process.

Angles I and II ( Figure 2c ), listed in Table 1 , corresponding to each group, were measured by two independent observers as described in the Methods section. Based on manual angle measurement, the ICC between the two independent observers was 0.99, which was considered excellent,^28^ showing the high reproducibility of the method. No statistical difference was found in both angles regarding the presence or absence of aneurysm growth, and a sample of nine individuals does not allow major statistical inferences. Nevertheless, the high rate of agreement between the observers may allow, with a higher number of patients analyzed, significant statistical associations in the near future. As an unprecedented study, it could be considered a proof of concept in terms of numerical research and a study of disruptive translational technology.

By comparing the flow field inside the aorta of nine patients, with a time span of at least one year, different coherent structures were observed. Toroidal structures with low velocities detached from the distal aortic wall were observed for the patients without aneurysm growth. For those with aneurysm growth, two types of structures were identified: hairpin-like structures and toroidal structures with high velocities.

Since the helicity distribution indicating two directions of rotation occurs in the patients with aneurysm growth, it is possible to infer that in these cases the flow is likely to be more disturbed. Another possible consideration is that the flow inside the aneurysm contains regions of strong recirculation, which are responsible for the formation of coherent structures in the aorta. Michel et al.^37^ have discussed that alterations in blood flow patterns can induce aneurysmal development on the outer curvature of the ascending aorta, due to high transverse wall shear stress and mechanical impedance on the convex side.

Biasetti et al.^38^ have correlated the presence of vortical structures with high shear stress in patients with abdominal aortic aneurysms. Here, vertical coherent structures can also be seen near the distal aortic wall, evolving in some cases to a hairpin-like structure. These differences in the flow structure of patients in the two different groups (with and without aneurysm growth) may indicate the possibility of an aortic aneurysm remodeling process.

One hypothesis to explain these findings is the transfer of stress to adventitia due to loss of elastin and collagen deposition, promoting continuous adaptation because of stress distribution and, consequently, changes in the shape of AsAA.^39^

There are some limitations to this research. Firstly, the CTA scans were performed by different physicians. As a result, the quality of the exams was not the same. This fact may have affected the accuracy of measurements and definition of the aortic shape. Secondly, the small size of the population studied limits any correlation between clinical patterns and AsAA growth. Thirdly, clinical data of two patients were not available, so they were not included in Table 2 . In addition, the present analysis was performed considering the most critical situation during the systole corresponding to the maximum flow rate in a cardiac cycle. Thus, the aorta was considered static at systolic peak and its elasticity was not considered.

Clearly, there are several factors that must be considered to improve the prognosis of patients with regards to aortic aneurysm growth, such as their age, previous diseases, and medicine intake. The angle between the aortic inlet and the brachiocephalic trunk could be an important feature to be considered, as well as the flow structure and the pressure distribution on the anterior wall, which can be numerically determined.

By the pressure distribution in the aortic wall, the mean pressure was observed to increase over 13% in all patients with aneurysm growth, while for those without pathological growth, the mean pressure decreased over 18% for all patients, except for one. This result indicates that this variable might influence the growth of the AsAA.

Future studies with a larger number of patients and longer follow-up are needed to precisely define the relationship between blood flow pattern and aortic remodeling process. The CFD could be a completely non-invasive tool to be used from CTA scans to estimate the growth trend of AsAA. The identification of different risk profiles among patients with AsAA could open the way for different medical care for these patients. All the above can lead to a step forward in personalized medicine and help to decipher the pathophysiology of this severe disease.

Considering that some flow characteristics could be associated with aneurysm growth, the study of the flow pattern in the ascending aorta could help predict the remodeling process of AsAA.

## Conclusions

This pilot study showed that CFD based on CTA may in the near future be a tool to help identify patterns of flow behavior associated with the AsAA remodeling process. Vortices were formed in the posterior region of the incident jet in the aortic wall, generating more complex structures for the aneurysm growth group. Consequently, there was an increase in the mean pressure in the anterior aortic wall between the CTA exams for the patients with aneurysm growth, while for those without aneurysm growth, this variable decreased.
